# Nursing Home Resident Symptomatology Triggering Transfer: Avoiding Unnecessary Hospitalizations

**DOI:** 10.1155/2012/495103

**Published:** 2012-10-03

**Authors:** Alyce S. Ashcraft, Jane Dimmitt Champion

**Affiliations:** ^1^School of Nursing, Texas Tech University Health Sciences Center, 3601 4th Street, Lubbock, TX 79430, USA; ^2^School of Nursing, The University of Texas at Austin, 1700 Red River Street, Austin, TX 78712, USA

## Abstract

The purpose of this study was to describe nursing home resident symptomatology and medical diagnoses associated with nursing home to hospital transfers. A retrospective chart review of documented transfers was conducted at a 120-bed, nonprofit urban Continuing Care Retirement Center nursing home facility located in the southwestern United States. The transferred residents (*n* = 101) had seventy different medical diagnoses prior to hospital transfer with hypertension, coronary artery disease, and congestive heart failure most frequently reported. Most frequently reported symptomatology included fatigue, lethargy or weakness, shortness of breath, and change in level of consciousness. Multiple symptomatology was indicative of a wide variety of medical diagnoses. The diagnoses and symptomatology recorded in this paper identify the importance of strategic planning concerning assessment and communication of common nursing home resident symptomatology and the importance of basic nursing and diagnostic procedures for prevention of potentially avoidable hospitalizations.

## 1. Introduction

 Potentially avoidable nursing home hospitalizations have become a focus for the United Stated Medicare and Medicaid System because of burgeoning costs totaling billions of dollars annually. One-third of nursing home residents are hospitalized each year. It is imperative to reduce avoidable hospital transfers to not only decrease cost to the health-care system but to also prevent the physical, psychological, and iatrogenic events that frequently manifest with hospital transfer [1]. Nursing home staff must be knowledgeable about the disease processes associated with advanced age, multiple comorbidities, and functional impairment to effectively and efficiently identify relevant symptomatology and communicate resident changes to avoid unnecessary hospitalizations [2]. Current nursing home staffing standards impact the process for reduction in unnecessary hospitalizations. Nursing home facilities are required to minimally provide licensed charge nurses (Licensed Vocational Nurses/LVN or Registered Nurses/RN) on each work shift [3]. The charge nurse managing the care of multiple patients is frequently an LVN, with activities of daily living provided by a Certified Nursing Assistant (CNA). An LVN has one year of education concerning basic nursing essentials and is responsible for providing safe nursing for individuals with predictable healthcare needs. The CNA has a minimum of 75 clock hours of training and is responsible for providing basic personal care to residents. The CNA reports resident changes to the LVN. Once changes are validated, the LVN reports findings to an RN (if available) or provider (physician, nurse practitioner, or physician assistant) for management. Limitations in education and experience of these two health care providers impact reduction of unnecessary hospitalization as evidence indicates increases in RN direct care time are strongly associated with better resident outcomes [3, 4].

Licensed Vocational Nurses are responsible for collecting and communicating data about resident status change to providers (physicians, nurse practitioners, physician's assistants) for timely intervention; however these nurses may not accurately associate symptomatology with specific disease processes. The CNA role is to discern resident status change and communicate this to a Licensed Vocational or Registered Nurse. Expedient identification of symptomatology, communication of resident status change, and implementation of recommended treatment provide the opportunity for nursing home versus hospital treatment of resident health conditions. Expedient identification of symptomatology is a first step toward reduction of avoidable hospitalizations. The purpose of this study was to describe nursing home resident symptomatology and medical diagnoses associated with nursing home to hospital transfers. This information is intended to augment understanding of the knowledge requirements for nursing home staff and as well as the importance of expedient interventions to reduce avoidable nursing home hospitalizations. 

Nursing home hospitalization rates reportedly vary and can be extremely high (9% to 59%) [5–7]. The percentages of hospitalizations that are potentially avoidable are reportedly even higher (55% to 67%) [5–7]. This indicates that nursing home residents are transferred for hospital care rather than treatment in the nursing home setting. Differentiating between residents who require hospital transfer and residents who may be effectively treated in the nursing home is imperative for cost-effective care. Factors associated with avoidable hospitalizations however are complex and include resident health, resident and family preferences, nursing home resources, and nursing home policies [5–7]. Resident health status is a primary factor in determining the need for hospitalization [8]. Hospitalization has been found to occur most frequently among physically disabled [9–12] as well as chronically ill nursing home residents [9, 10, 13–17]. Physical restraints, pressure ulcers, feeding tubes, new medications, and catheter use are also associated with nursing home resident hospitalization [4, 10, 13]. Hospital transfer increases have also been identified in situations including a lack of support to residents and their families about end-of-life care, lack of familiarity with residents by health care providers covering nights and weekends, and perceived inadequate care by overburdened staff [18–20]. 

 Organizational factors that decrease nursing home resident hospitalization include effective communication between nursing staff and physicians regarding resident condition, the employment of nurse practitioners or physician assistants in the nursing home setting, provider access to resident medical history, laboratory results, and electrocardiograms, availability of treatment modalities such as intravenous therapy and respiratory therapy, and CNA training programs within the nursing home facility [12, 20–22]. Resources used to prevent nursing home resident hospitalization not only decrease healthcare costs but also prevent stress and exposure to iatrogenic illness. This study describes nursing home resident symptomatology and medical diagnoses associated with hospital transfer of residents in a nursing home setting. This information has the potential to assist in recognition of resident symptoms and subsequent diagnoses requiring early intervention to reduce adverse outcomes.

## 2. Methods 

A retrospective chart review process was utilized for this descriptive study. After receiving Institutional Review Board approval, all charts of residents transferred from the nursing home to a hospital from September 1, 2007 to September 1, 2008 were requested from the Medical Records Director and reviewed by either the primary investigator or the research assistant. The nursing home setting was a 120-bed combination nursing home and skilled nursing facility (a licensed health care facility that cares for individuals with temporary or permanent complex health problems). 

Data were extracted from the chart using a Resident Characteristics Form. Data described resident demographics (date of admission to the nursing home, prior location on admission to the nursing home, payer source, gender, age, marital status, education, number of transfers to the hospital during the timeframe of the study), resident symptoms documented at transfer by the LVN, procedures performed prior to transfer, documented medical diagnoses from the day of nursing home admission, and new medical diagnoses upon return to the nursing home. 

Statistical analysis was conducted using SPSS 14.0 and included means, medians, and ranges. Chi square analyses were completed to assess statistical differences between groups. Descriptive statistics were used for analysis of symptomatology and medical diagnoses. Symptomatology as recorded was grouped using commonly accepted medical terminology.

## 3. Results

 The medical chart review identified the following characteristics of 101 residents transferred from the nursing home to hospitals for treatment. Residents transferred to the acute care environment were predominately female (*n* = 72, 71%) and widowed (*n* = 72, 71%) with Medicare as the primary payer source for 78% (*n* = 78). The majority of residents had been admitted to the nursing home within the last two years (*n* = 24 males, 83% of males; *n* = 60 females, 83% of females) from hospitals (*n* = 80; 79%), assisted living (*n* = 4; 4%), or other community (*n* = 13; 11%) settings. Nursing home transfers to the hospital occurred throughout the year with the largest number occurring in February, December, and March. 

Residents received multiple (*n* = 70) different medical diagnoses prior to hospital transfer. Seventy different medical diagnoses were documented prior to hospital transfer. The most frequent diagnoses included hypertension (*n* = 57; 57%), coronary artery disease (*n* = 29, 29%), congestive heart failure (*n* = 29; 29%), diabetes mellitus (*n* = 17; 17%), hypothyroid (*n* = 17; 17%), dementia (*n* = 15; 15%), gastroesophageal reflux disease (*n* = 14; 14%), chronic obstructive pulmonary disease (*n* = 13; 13%), atrial fibrillation (*n* = 12; 12%), and recovery from open reduction internal fixation of the hip (*n* = 12; 12%) (Table 1). Comparisons of these diagnoses were made by gender. A significant difference for hypertension was found with more males than females receiving this diagnosis upon admission (males 72%, females 50%, *P* = .04). 

 Seventy-two symptom categories were identified. Symptomatology identified 10 or more times included fatigue, lethargy, tiredness, or weakness (*n* = 23, 23%), shortness of breath (*n* = 20, 20%), change in level of consciousness (*n* = 19, 19%), complaint of muscle or bone pain (*n* = 19, 19%), elevated blood pressure (*n* = 17, 17%), decreased oxygenation (*n* = 17, 17%), edema/swollen body part/hematoma (*n* = 17, 17%), chest pain, pressure, or tightness (*n* = 16, 16%), elevated heart rate (*n* = 14, 14%), nausea or vomiting (*n* = 14, 14%), decreased blood pressure (*n* = 13, 13%), and elevated temperature (*n* = 13, 13%) (Table 1). Comparisons of symptoms were made by gender. A significant difference was found for complaint of muscle or bone pain by gender (males 31%, females 14%, *P* = .046). 

Prior to transfer, nursing home staff carried out 17 different procedures. The seven most common procedures included vital signs (*n* = 52, 51%), medication administration (po or inhalation; *n* = 32, 32%), oxygen by nasal cannula (*n* = 25, 25%), X-rays (*n* = 22, 22%), ECG (*n* = 6, 6%), oxygen saturation (*n* = 5, 5%), and Doppler studies (arterial or venous) (*n* = 5, 5%) (Table 1). 

 Of new medical diagnoses (*n* = 68, 68%) documented upon return to the nursing home, pneumonia (*n* = 13, 13%), urinary tract infection (*n* = 13, 13%), dehydration (*n* = 9, 9%), congestive heart failure (*n* = 8, 8%), chronic obstructive pulmonary disease (*n* = 7, 7%), end-stage renal disease (*n* = 6, 6%), atrial fibrillation (*n* = 5, 5%), any fracture (*n* = 5, 5%), open reduction internal fixation hip (*n* = 5, 5%), and cut/contusion/hematoma (*n* = 4, 4%) were most common (Table 1). Comparisons of these new medical diagnoses were made by gender. No differences in new medical diagnoses were found with comparisons by gender.

## 4. Discussion

This study identified common medical diagnoses before and after hospital transfer as well as common signs and symptoms prior to transfer and common procedures completed prior to transfer. As a result, this study provides information about the knowledge and skills needed by nursing home staff to avoid unnecessary resident hospitalization. 

Residents admitted to the nursing home typically have multiple medical diagnoses requiring ongoing management. A key to resident health is nursing staff familiarity with individual resident norms in order to facilitate identification of potential resident problems and necessity for provider notification. The findings from this study are similar to others regarding common diagnoses of nursing home residents and diagnoses requiring their hospitalization [23]. The most common new diagnoses residents received upon return to the nursing home in this study were pneumonia, urinary tract infection, and dehydration. These diagnoses are associated with fatigue, shortness of breath, change in level of consciousness, chest pain, elevated heart rate, and chest pain. These symptoms are associated with a myriad of medical diagnoses (Table 1). The interpretation of these diagnoses is dependent upon nurse and provider understanding of a resident's complex history. Trending of data presumes multifaceted reasoning skills that are required from RNs and providers; however this skill is beyond the LVN scope of predictable health care needs [4, 24].

Though resident status changes are often difficult to assess, they can be even more difficult to communicate. This is apparent in the symptoms documented prior to hospital transfer (Table 1). Checklist documentation to facilitate communication of multiple symptoms identified in this retrospective chart review may improve accuracy and promptness of diagnosis and interventions by giving a more complete assessment of the resident for the provider, subsequently reducing the number of unnecessary hospital transfers. As electronic medical records are incorporated into long-term care, documentation templates may contain prompts for identification and communication of symptomatology identified in this study. 

Consistent assignment of nursing home resident care for direct care staff, in particular CNAs, is not always possible. Prompts to communicate pertinent data to LVNs or RNs concerning resident status are indicated. Continuing education is essential for all nursing home direct care personnel to include an understanding of geriatric syndromes (e.g., delirium, falls, frailty, dizziness, syncope, urinary incontinence) to facilitate data collection and effective communication of resident change. This may be achieved through debriefing following resident transfer, discussion of individual resident case scenarios, and competency training using simulation. 

Nursing procedures utilized in the nursing home setting prior to hospital transfer included vital signs, medication administration by multiple routes, oxygen administration, X-rays, electrocardiogram, oxygen saturation, and Doppler studies. These procedures and diagnostic findings provided information for critical assessment and interpretation concerning whether or not residents could be treated in the nursing home or required transfer. Other procedures that may be done prior to initiating transfer include obtaining blood analyses, beginning intravenous fluids, and inserting urinary catheters. These procedures need to be added to the list of frequently performed procedures as they may also prevent unnecessary transfer to hospital settings. Poor communication as well as suboptimal implementation of procedures and interpretation of data, contributes significantly to unnecessary hospital transfers [20, 25, 26]. 

## 5. Conclusions

Although the study findings are from one institution and the sample size is small, the findings support those previously identified with regard to common diagnoses associated with nursing home resident to hospital transfer, and are extended these findings by identifying symptoms commonly triggering transfer. The study findings emphasize the need for nursing home personnel understanding of basic disease processes and chronic disease management. Administrative and clinical strategies are indicated to ensure timely identification of resident problems and accurate communication of resident status among nursing staff. Nursing staff require competence for performance of a variety of procedures within the nursing home setting to facilitate diagnosis and treatment in order to reduce unnecessary hospital transfers.

## Figures and Tables

**Table 1:** Medical diagnoses leading to symptoms leading to procedures.

Medical diagnoses prior to transfer	Symptoms prior to transfer	Procedures prior to transfer
Hypertension	Fatigue, lethargy, tiredness, weakness	Vital signs
Coronary artery disease	Shortness of breath	Medication administration
Congestive heart failure	Change in level of consciousness	Oxygen by nasal cannula
Diabetes mellitus	Complaint of muscle or bone pain	X-rays
Hypothyroid	Elevated blood pressure	Electrocardiogram
Dementia	Decreased oxygenation	Oxygen saturation
Gastroesophageal reflux disease	Edema, swollen body part, hematoma	Doppler studies
Chronic obstructive pulmonary disease	Chest pain, pressure, or tightness	
Atrial fibrillation	Elevated heart rate	
Recovery from open reduction internal fixation of the hip	Nausea or vomiting	
	Decreased blood pressure	
	Elevated temperature	

New medical diagnoses after transfer		

Pneumonia		
Urinary tract infection		
Dehydration		
Congestive heart failure		
Chronic obstructive pulmonary disease		
End-stage renal disease		
Atrial fibrillation		
Any fracture		
Open reduction internal fixation of the hip		
Cut, contusion, hematoma		

## References

[1] Ouslander JG, Lamb G, Tappen R (2011). Interventions to reduce hospitalizations from nursing homes: evaluation of the INTERACT II collaborative quality improvement project. *Journal of the American Geriatrics Society*.

[2] Wolff JL, Starfield B, Anderson G (2002). Prevalence, expenditures, and complications of multiple chronic conditions in the elderly. *Archives of Internal Medicine*.

[3] Castle NG (2008). Nursing home caregiver staffing levels and quality of care: a literature review. *Journal of Applied Gerontology*.

[4] Horn SD, Buerhaus P, Bergstrom N, Smout RJ (2005). RN staffing time and outcomes of long-stay nursing home residents. *American Journal of Nursing*.

[5] Grabowski DC, Stewart KA, Broderick SM, Coots LA (2008). Predictors of nursing home hospitalization—a review of the literature. *Medical Care Research and Review*.

[6] Ouslander JG, Lamb G, Perloe M (2010). Potentially avoidable hospitalizations of nursing home residents: frequency, causes, and costs. *Journal of the American Geriatrics Society*.

[7] Walker JD, Teare GF, Hogan DB, Lewis S, Maxwell CJ (2009). Identifying potentially avoidable hospital admissions from Canadian long-term care facilities. *Medical Care*.

[8] Muenchberger H, Kendall E (2010). Predictors of preventable hospitalization in chronic disease: priorities for change. *Journal of Public Health Policy*.

[9] Freiman MP, Murtaugh CM (1993). The determinants of the hospitalization of nursing home residents. *Journal of Health Economics*.

[10] Fried TR, Mor V (1997). Frailty and hospitalization of long-term stay nursing home residents. *Journal of the American Geriatrics Society*.

[11] Gillen P, Spore D, Mor V, Freiberger W (1996). Functional and residential status transitions among nursing home residents. *Journals of Gerontology A*.

[12] Konetzka RT, Spector W, Shaffer T (2004). Effects of nursing home ownership type and resident payer source on hospitalization for suspected pneumonia. *Medical Care*.

[13] Carter MW (2003). Factors associated with ambulatory care-sensitive hospitalizations among nursing home residents. *Journal of Aging and Health*.

[14] Carter MW, Porell FW (2006). Nursing home performance on select publicly reported quality indicators and resident risk of hospitalization: grappling with policy implications. *Journal of Aging and Social Policy*.

[15] Intrator O, Castle NG, Mor V (1999). Facility characteristics associated with hospitalization of nursing home residents: results of a national study. *Medical Care*.

[16] Mor V, Papandonatos G, Miller SC (2005). End-of-life hospitalization for African American and non-latino white nursing home residents: variation by race and a facility’s racial composition. *Journal of Palliative Medicine*.

[17] Tresch DD, Simpson WM, Burton JR (1985). Relationship of long-term and acute-care facilities. The problem of patient transfer and continuity of care. *Journal of the American Geriatrics Society*.

[18] Bottrell MM, O’Sullivan JF, Robbins MA, Mitty EL, Mezey MD (2001). Transferring dying nursing home residents to the hospital: DON perspectives on the nurse’s role in transfer decisions. *Geriatric Nursing*.

[19] Buchanan JL, Murkofsky RL, O’Malley AJ (2006). Nursing home capabilities and decisions to hospitalize: a survey of medical directors and directors of nursing. *Journal of the American Geriatrics Society*.

[20] Young Y, Barhydt NR, Broderick S, Colello AD, Hannan EL (2010). Factors associated with potentially preventable hospitalization in nursing home residents in New York state: a survey of directors of nursing. *Journal of the American Geriatrics Society*.

[21] Grabowski DC, O’Malley AJ, Barhydt NR (2007). The costs and potential savings associated with nursing home hospitalizations. *Health Affairs*.

[22] Intrator O, Zinn J, Mor V (2004). Nursing home characteristics and potentially preventable hospitalizations of long-stay residents. *Journal of the American Geriatrics Society*.

[23] Byrd L (2009). Reducing avoidable hospitalizations in nursing could save $1 billion annually—so why delay?. *Geriatric Nursing*.

[24] Hutt E, Ecord M, Eilertsen TB, Frederickson E, Kramer AM (2002). Precipitants of emergency room visits and acute hospitalization in short-stay medicare nursing home residents. *Journal of the American Geriatrics Society*.

[25] McMahan EM, Hoffman K, McGee GW (1994). Physician-nurse relationships in clinical settings: a review and critique of the literature, 1966–1992. *Medical Care Review*.

[26] Robinson FP, Gorman G, Slimmer LW, Yudkowsky R (2010). Perceptions of effective and ineffective nurse-physician communication in hospitals. *Nursing Forum*.

